# Colonization with antibiotic-susceptible strains protects against methicillin-resistant *Staphylococcus aureus *but not vancomycin-resistant enterococci acquisition: a nested case-control study

**DOI:** 10.1186/cc10445

**Published:** 2011-09-14

**Authors:** Susan S Huang, Rupak Datta, Sheryl Rifas-Shiman, Ken Kleinman, Hilary Placzek, Julie D Lankiewicz, Richard Platt

**Affiliations:** 1 Division of Infectious Diseases and Health Policy Research Institute, University of California Irvine School of Medicine, 100 Theory, Ste 110, Irvine, CA 92697, USA; 2 Channing Laboratory, Brigham and Women's Hospital and Harvard Medical School, 181 Longwood Avenue, Boston, MA 02115 USA; 3 Department of Population Medicine, Harvard Pilgrim Health Care Institute and Harvard Medical School, 133 Brookline Avenue, Boston, MA 02215, USA; 4 Department of Clinical and Population Health Research, University of Massachusetts Medical School, 55 Lake Avenue North, Worcester, MA 01655, USA

## Abstract

**Introduction:**

Harboring sensitive strains may prevent acquisition of resistant pathogens by competing for colonization of ecological niches. Competition may be relevant to decolonization strategies that eliminate sensitive strains and may predispose to acquiring resistant strains in high-endemic settings. We evaluated the impact of colonization with methicillin-sensitive *Staphylococcus aureus *(MSSA) and vancomycin-sensitive enterococci (VSE) on acquisition of methicillin-resistant *Staphylococcus aureus *(MRSA) and vancomycin-resistant enterococci (VRE), respectively, when controlling for other risk factors.

**Methods:**

We conducted a nested case-control study of patients admitted to eight ICUs performing admission and weekly bilateral nares and rectal screening for MRSA and VRE, respectively. Analyses were identical for both pathogens. For MRSA, patients were identified who had a negative nares screen and no prior history of MRSA. We evaluated predictors of MRSA acquisition, defined as a subsequent MRSA-positive clinical or screening culture, compared to those with a subsequent MRSA-negative nares screen within the same hospitalization. Medical records were reviewed for the presence of MSSA on the initial MRSA-negative nares screen, demographic and comorbidity information, medical devices, procedures, antibiotic utilization, and daily exposure to MRSA-positive patients in the same ward. Generalized linear mixed models were used to assess predictors of acquisition.

**Results:**

In multivariate models, MSSA carriage protected against subsequent MRSA acquisition (OR = 0.52, CI: 0.29, 0.95), even when controlling for other risk factors. MRSA predictors included intubation (OR = 4.65, CI: 1.77, 12.26), fluoroquinolone exposure (OR = 1.91, CI: 1.20, 3.04), and increased time from ICU admission to initial negative swab (OR = 15.59, CI: 8.40, 28.94). In contrast, VSE carriage did not protect against VRE acquisition (OR = 1.37, CI: 0.54, 3.48), whereas hemodialysis (OR = 2.60, CI: 1.19, 5.70), low albumin (OR = 2.07, CI: 1.12, 3.83), fluoroquinolones (OR = 1.90, CI: 1.14, 3.17), third-generation cephalosporins (OR = 1.89, CI: 1.15, 3.10), and increased time from ICU admission to initial negative swab (OR = 15.13, CI: 7.86, 29.14) were predictive.

**Conclusions:**

MSSA carriage reduced the odds of MRSA acquisition by 50% in ICUs. In contrast, VSE colonization was not protective against VRE acquisition. Studies are needed to evaluate whether decolonization of MSSA ICU carriers increases the risk of acquiring MRSA when discharging patients to high-endemic MRSA healthcare settings. This may be particularly important for populations in whom MRSA infection may be more frequent and severe than MSSA infections, such as ICU patients.

## Introduction

Methicillin-resistant *Staphylococcus aureus *(MRSA) and vancomycin-resistant enteroccoci (VRE) cause substantial morbidity and mortality in hospitalized populations [1-6]. Patients acquiring MRSA or VRE incur significant risks of subsequent infection. Up to 11% of MRSA-colonized in-patients develop MRSA disease during their hospital stay, and this risk can approach 30% in the critically ill [7-9]. Similarly, 19% of VRE-colonized patients in the intensive care unit (ICU) develop VRE infection during hospitalization, and this risk can approach 32% in those with transplants or cancer [10-12]. Risks of MRSA and VRE infection after colonization also extend into the post-discharge setting. We previously found that 29% of MRSA carriers and 8% of VRE carriers developed invasive disease within 18 months, with post-discharge MRSA and VRE infections often requiring readmission [13-15].

Importantly, compared to MSSA colonization, MRSA colonization is associated with a four-fold increase in infection risk [16]. Even after controlling for host risk factors, it has been shown that MRSA colonized-patients are more likely to develop subsequent infection compared to MSSA-colonized patients [17]. These differential risks of infection following colonization are important since MRSA bacteremia is associated with greater attributable morbidity and mortality compared to MSSA bacteremia [2,3,18]. Moreover, the increased mortality associated with MRSA versus MSSA extends up to three months after hospital discharge [19].

Given these risks, many studies have evaluated predictors of MRSA and VRE acquisition to improve infection prevention methods. Most predictors of MRSA acquisition have related to host comorbidities, hospital factors, and antimicrobial use. For instance, diabetes, hemodialysis, ulcers, trauma, ICU stays, admission to surgical ICUs, and antibiotic exposure are known to predispose to MRSA colonization [20-26]. Although VRE carriage more commonly occurs in the critically ill, similar risk factors exist for VRE acquisition including immunosuppression, neutropenia, hematologic malignancies, ICU admission, and antibiotic exposure [27-32]. Environmental contamination has also been associated with MRSA and VRE acquisition [33-39].

Although predictors of MRSA and VRE colonization have been well documented, less is known about protective factors. It has been hypothesized that methicillin-sensitive *Staphylococcus aureus *(MSSA) and other antibiotic-sensitive bacteria may protect against MRSA acquisition by competing for colonization of the anterior nares [40]. Competition may be relevant to decolonization strategies that may eliminate MSSA and predispose to MRSA acquisition in high endemic settings such as ICUs and nursing homes. Therefore, we assessed whether MSSA or vancomycin-sensitive enterococci (VSE) colonization reduces the risk of MRSA and VRE acquisition, respectively.

## Materials and methods

We conducted a retrospective nested case-control study of patients admitted to eight adult ICUs between 1 September 2003 and 30 April 2005 at a 750-bed academic medical center in Boston, Massachusetts, who were not previously known to have MRSA or VRE and who had MRSA-negative or VRE-negative surveillance cultures upon ICU admission. All ICUs performed high-compliance admission and weekly surveillance bilateral nares cultures for MRSA and rectal cultures for VRE, providing a systematic method to distinguish between imported and incident cases during endemic conditions. ICUs included medical, cardiac, general surgery, burn/trauma, cardiac surgery (two units), thoracic surgery, and neurosurgery units, and each had a 10-bed capacity. This study was approved by the Institutional Review Board at the Brigham and Women's Hospital. A waiver of informed consent was granted.

**Figure 1:**
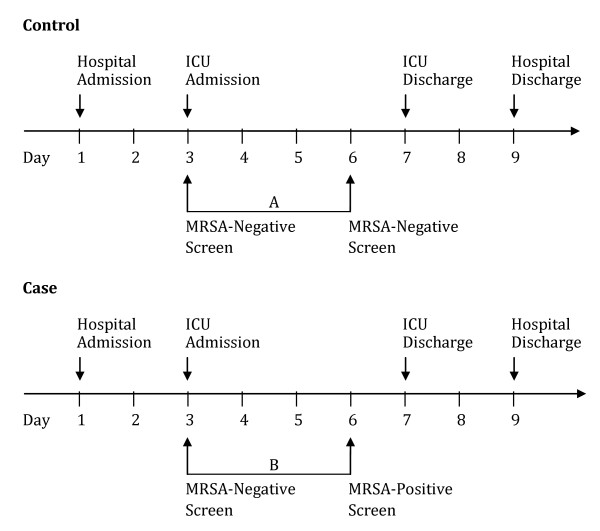
**Identification of methicillin-resistant *Staphylococcus aureus *(MRSA) cases and controls following intensive care unit (ICU) admission**. All patients were required to have an initial MRSA-negative bilateral nares screening culture. Controls had a subsequent MRSA-negative screening culture (Interval **A**) whereas cases had a subsequent MRSA-positive screening or clinical culture (Interval **B**).

We report details regarding the MRSA cohort, but data collection and analyses were performed identically for MRSA and VRE. We obtained census information detailing ICU patients and occupancy dates during the study period. We identified all patients who had an MRSA-negative bilateral nares screening culture and no prior history of MRSA using microbiology laboratory and infection control records dating back to 1987. We then identified patients who had either (1) a subsequent MRSA-negative bilateral nares screening culture (control) or (2) a subsequent MRSA-positive clinical or screening culture (case) within the same hospital stay (Figure 1). Subsequent MRSA screening generally reflected routine ICU protocol for weekly screening of ICU patients on a predetermined weekday. From this cohort, we selected all cases and a random sample of controls, and variables associated with MRSA acquisition were evaluated. All MRSA-negative surveillance cultures were routinely evaluated for the presence of MSSA, thereby enabling the systematic identification of MSSA colonization among cases and controls.

For all MRSA and VRE cases and controls, we collected demographic and comorbidity information based on *International Classification of Diseases, Ninth Revision, Clinical Modification *codes from same-hospital admissions within one year prior to the initial negative surveillance culture. Measured comorbidities included diabetes, end-stage renal disease, end-stage liver disease, solid cancers, and hematologic malignancies, which were confirmed by medical chart review. Malignancies were recorded only if there was evidence of treatment in the preceding year. We also identified risk factors for MRSA and VRE infection during the two weeks prior to the initial negative surveillance culture through the period encompassing the subsequent negative or positive surveillance or clinical culture. Risk factors included active wounds and rashes, recent surgery, and non-surgical procedures including intubation, bronchoscopy, and the placement of central lines, drains, or tubes. During this time, we additionally collected laboratory indicators of renal insufficiency (creatinine > 2) and poor nutrition (albumin < 2). For the period of time encompassing two months prior to the initial negative surveillance culture until the time of the subsequent negative or positive surveillance or clinical culture, we also recorded antibiotic administration data from the following classes: narrow and broad-spectrum penicillins, first, second and third generation cephalosporins, fluoroquinolones, carbapenems, aminoglycosides, macrolides, and anti-MRSA (vancomycin, linezolid, synercid, daptomycin, tigecycline), other MRSA (doxycycline, bactrim, rifampin), anti-VRE (linezolid, synercid, daptomycin, tigecycline), or other VRE (doxycycline, nitrofurantoin)antibiotics. Time from ICU admission to the initial MRSA-negative culture was also assessed.

Additionally, we assessed colonization pressure, defined as the sum of the daily number of same ward MRSA-positive or VRE-positive patients to which patients were exposed between the initial negative culture and the subsequent positive or negative culture for MRSA or VRE. In addition to evaluating colonization pressure as a total number of daily exposures, we additionally evaluated colonization pressure as a density of exposures divided across the number of days spanned by the initial negative culture and the subsequent positive or negative culture for MRSA or VRE.

Potential predictors of MRSA or VRE acquisition were initially assessed using χ2 bivariate tests. Variables significant in bivariate testing at a level of α < 0.2 were entered into multivariate generalized linear mixed models (GLIMMIX, version 9.1; SAS, SAS Institute, Cary, NC, USA). All bivariate and multivariate analyses accounted for clustering by ICU ward. The primary independent variables of MSSA and VSE carriage were forced into the final model. All other final model variables were retained at α = 0.05.

## Results

Across a 20-month period, a total of 8,203 patients had 11,528 ICU room stays. Among them, 809 patients and 658 patients were already MRSA and VRE carriers on ICU admission, leaving 7,629 and 7,806 patients eligible for MRSA and VRE acquisition, respectively. Of these, 244 and 227 patients acquired MRSA and VRE based upon a positive culture in patients with a negative surveillance culture and no prior history of MRSA or VRE, respectively. Cases were compared to a random sample of 250 controls for each pathogen. Medical records for two controls were unavailable, resulting in a total of 248 MRSA-negative controls and 248 VRE-negative controls.

Descriptive characteristics of MRSA and VRE cases and controls are shown in Tables 1 and 2, respectively. Patient characteristics were similar across MRSA and VRE cohorts, with half being over 65 years old, nearly one-third having diabetes and one-quarter with a solid cancer. The vast majority had recent surgeries or non-surgical procedures. Compared to controls, MRSA cases had a significantly longer mean time from ICU admission to initial MRSA-negative swab (mean 4.9 versus 1.4 days, *P *< 0.001). Similar results were found for VRE (5.5 versus 1.3 days, *P *< 0.001).

**Table 1:** Bivariate assessment of characteristics associated with methicillin-resistant *Staphylococcus aureus *(MRSA) acquisition among cases and controls

Variable	Controls ^a^ N (%)	Cases ^a^ N (%)	Odds ratio ^b^ (95% CI)	*P*-value
Total	248 (100%)	244 (100%)		
Age				0.47
< 45	40 (16%)	38 (16%)	1.0, reference	
45 to < 55	31 (13%)	26 (11%)	0.93 (0.46, 1.88)	
55 to < 65	59 (24%)	54 (22%)	1.12 (0.62, 2.04)	
65 to < 75	54 (22%)	68 (28%)	1.56 (0.85, 2.85)	
75+	64 (26%)	58 (24%)	1.10 (0.60, 2.01)	
Male gender	146 (59%)	140 (57%)	1.11 (0.77, 1.59)	0.58
Comorbidities				
Diabetes mellitus	71 (29%)	70 (29%)	1.07 (0.71, 1.61)	0.76
End-stage renal disease	12 (5%)	20 (8%)	1.67 (0.79, 3.53)	0.18
End-stage liver disease	6 (2%)	12 (5%)	2.20 (0.80, 6.08)	0.13
Solid cancer	63 (25%)	64 (26%)	1.07 (0.67, 1.69)	0.78
Hematologic malignancy	13 (5%)	9 (4%)	0.71 (0.29, 1.75)	0.46
ICU Type				0.26
Medical	82 (33%)	61 (25%)	0.67 (0.33, 1.35)	
Surgical	166 (67%)	183 (75%)		
ICU admit to negative swab				< .0001
1 day	183 (74%)	82 (34%)	1.0, reference	
2 days	50 (20%)	41 (17%)	1.81 (1.11, 2.97)	
≥ 3 days	15 (6%)	121 (50%)	17.82 (9.77, 32.49)	
Active wound	181 (73%)	193 (79%)	1.20 (0.76, 1.89)	0.43
Active rash	30 (12%)	33 (14%)	1.18 (0.69, 2.02)	0.55
Surgical procedures ^c^	207 (83%)	196 (80%)	0.72 (0.45, 1.17)	0.19
Non-surgical procedures ^c^				
Intubation	213 (86%)	238 (98%)	6.09 (2.48, 14.94)	< .0001
Central line	192 (77%)	214 (88%)	2.10 (1.27, 3.48)	0.004
Arterial line	226 (91%)	232 (95%)	1.78 (0.84, 3.78)	0.13
Chest tube	94 (38%)	86 (35%)	0.87 (0.54, 1.40)	0.57
Surgical drain	108 (44%)	98 (40%)	0.85 (0.57, 1.25)	0.41
Labs ^c^				
Albumin < 2	21 (9%)	34 (14%)	1.60 (0.89, 2.88)	0.11
Creatinine > 2	52 (21%)	68 (28%)	1.54 (0.99, 2.37)	0.05
Colonization pressure				0.08
0	9 (4%)	15 (6%)	1.33 (0.52, 3.38)	
1 to < 4	72 (29%)	51 (21%)	0.54 (0.33, 0.90)	
4 to < 8	66 (27%)	54 (22%)	0.64 (0.39, 1.05)	
8 to < 12	42 (17%)	42 (17%)	0.76 (0.44, 1.33)	
12+	59 (24%)	82 (34%)	1.0, reference	
Sensitive strain carrier ^d^	49 (20%)	30 (12%)	0.55 (0.33, 0.91)	0.02
Antibiotic utilization ^c^				
Aminoglycoside	33 (13%)	24 (10%)	0.71 (0.40, 1.26)	0.24
Clindamycin	20 (8%)	18 (7%)	0.94 (0.48, 1.85)	0.87
Macrolide	26 (10%)	26 (11%)	1.07 (0.59, 1.93)	0.83
Fluoroquinolone	146 (59%)	193 (79%)	2.91 (1.91, 4.42)	< .0001
First generation Cephalosporin	101 (41%)	107 (44%)	0.97 (0.65, 1.46)	0.89
Second generation Cephalosporin	3 (1%)	6 (2%)	1.93 (0.47, 8.03)	0.36
Third generation Cephalosporin	72 (29%)	88 (36%)	1.49 (1.01, 2.21)	0.05
Broad spectrum penicillin	0 (0%)	6 (2%)		
Carbapenem	6 (2%)	11 (5%)	2.13 (0.76, 5.96)	0.15
Anti-MRSA antibiotics ^e^	159 (64%)	182 (75%)	1.92 (1.26, 2.92)	0.002
Other MRSA antibiotics ^f^	10 (4%)	4 (2%)	0.40 (0.12, 1.31)	0.13

**^a^**Cases and controls had a negative bilateral nares surveillance swab and no prior history of MRSA upon intensive care unit admission

^b^Accounted for clustering by intensive care unit ward

^c^Assessed during the two weeks prior to the initial negative surveillance culture through the period encompassing the subsequent negative or positive surveillance or clinical culture for MRSA

^d^Sensitive strain refers to methicillin-sensitive *Staphyloccocus aureus*

^e^Anti-MRSA antibiotics include vancomycin, linezolid, synercid, daptomycin, and tigecycline

^f^Other MRSA antibiotics include doxycycline, bactrim, and rifampin

CI, confidence interval.

**Table 2:** Bivariate assessment of characteristics associated with vancomycin-resistant enterococcus (VRE) acquisition among cases and controls

Variable	Controls ^a^ N (%)	Cases ^a^ N (%)	Odds ratio ^b^ (95% CI)	*P *value
Total	248 (100%)	227 (100%)		
Age				0.98
< 45	24 (10%)	20 (9%)	1.0, reference	
45 to < 55	35 (14%)	31 (14%)	0.95 (0.43, 2.08)	
55 to < 65	51 (21%)	54 (24%)	1.13 (0.55, 2.35)	
65 to < 75	62 (25%)	58 (26%)	1.01 (0.49, 2.08)	
75+	76 (31%)	64 (28%)	0.97 (0.48, 1.98)	
Male gender	127 (51%)	139 (61%)	0.72 (0.49, 1.04)	0.08
Comorbities				
Diabetes mellitus	72 (29%)	81 (36%)	1.43 (0.95, 2.13)	0.08
End-stage renal disease	15 (6%)	29 (13%)	2.36 (1.21, 4.60)	0.01
End-stage liver disease	11 (4%)	8 (4%)	0.79 (0.31, 2.05)	0.63
Solid cancer	55 (22%)	64 (28%)	1.19 (0.74, 1.89)	0.47
Hematologic malignancy	15 (6%)	18 (8%)	1.16 (0.56, 2.43)	0.69
ICU type				0.77
Medical	92 (37%)	75 (33%)	0.88 (0.37, 2.06)	
Surgical	156 (63%)	152 (67%)		
ICU admit to negative swab				< .0001
1 day	186 (75%)	79 (35%)	1.0, reference	
2 days	48 (19%)	30 (13%)	1.46 (0.85, 2.52)	
≥ 3 days	14 (6%)	118 (52%)	18.92 (10.23, 34.97)	
Active wound	174 (70%)	184 (81%)	1.74 (1.07, 2.83)	0.03
Active rash	21 (8%)	40 (18%)	2.16 (1.22, 3.83)	0.01
Surgical procedures ^c^	218 (88%)	175 (77%)	0.42 (0.25, 0.71)	0.001
Non-surgical procedures ^c^				
Intubation	210 (85%)	214 (94%)	2.47 (1.25, 4.90)	0.01
Central line	200 (81%)	212 (94%)	2.99 (1.60, 5.62)	0.001
Arterial line	222 (90%)	211 (93%)	1.44 (0.73, 2.83)	0.29
Chest tube	97 (39%)	93 (41%)	0.79 (0.49, 1.27)	0.32
Surgical drain	106 (43%)	74 (33%)	0.61 (0.40, 0.93)	0.02
Labs ^c^				
Albumin < 2	28 (11%)	56 (25%)	2.39 (1.43, 3.98)	0.001
Creatinine > 2	74 (30%)	99 (44%)	2.03 (1.35, 3.05)	0.001
Colonization pressure				< .0001
0	29 (12%)	10 (4%)	0.17 (0.07, 0.38)	
1 to < 4	70 (28%)	21 (9%)	0.15 (0.08, 0.27)	
4 to < 8	58 (24%)	48 (21%)	0.40 (0.24, 0.67)	
8 to < 12	41 (17%)	43 (19%)	0.50 (0.29, 0.87)	
12+	50 (20%)	105 (46%)	1.0, reference	
Sensitive strain carrier ^d^	14 (6%)	14 (6%)	1.08 (0.50, 2.34)	0.85
Antibiotic utilization ^c^				
Aminoglycoside	24 (10%)	43 (19%)	2.27 (1.31, 3.93)	0.004
Clindamycin	14 (6%)	22 (10%)	1.90 (0.93, 3.88)	0.08
Macrolide	14 (6%)	30 (13%)	2.91 (1.47, 5.76)	0.002
Fluoroquinolone	141 (57%)	182 (81%)	2.94 (1.91, 4.50)	< .0001
First generation Cephalosporin	92 (37%)	68 (30%)	0.68 (0.45, 1.04)	0.08
Second generation Cephalosporin	8 (3%)	6 (3%)	0.72 (0.24, 2.15)	0.56
Third generation Cephalosporin	55 (22%)	107 (48%)	3.33 (2.20, 5.05)	< .0001
Broad spectrum penicillin	1 (0.40%)	3 (1%)	2.56 (0.26, 25.24)	0.42
Carbapenem	9 (4%)	21 (9%)	2.56 (1.13, 5.79)	0.02
Anti-VRE antibiotics ^e^	3 (1%)	9 (4%)	3.66 (0.95, 14.03)	0.06
Other VRE antibiotics ^f^	2 (0.81%)	3 (1%)	2.01 (0.32, 12.64)	0.46

^a^Cases and controls had a negative rectal surveillance swab and no prior history of VRE upon intensive care unit admission

^b^Accounted for clustering by intensive care unit ward

^c^Assessed during the two weeks prior to the initial negative surveillance culture through the period encompassing the subsequent negative or positive surveillance or clinical culture for VRE

^d^Sensitive strain refers to vancomycin-sensitive enterococci

^e^Anti-VRE antibiotics include linezolid, synercid, daptomycin, and tigecycline

^f^Other VRE antibiotics include doxycycline, nitrofurantoin

CI, confidence interval.

Table 1 further lists variables associated with MRSA acquisition in bivariate testing. Harboring MSSA was protective, while increased time from ICU admission to initial MRSA-negative swab, intubation, presence of a central line, fluoroquinolone utilization, and anti-MRSA antibiotic utilization were significantly associated with MRSA acquisition. In generalized linear mixed models, only MSSA carriage, intubation, fluoroquinolone utilization, and time from ICU admission to initial MRSA-negative swab remained associated with MRSA acquisition (Table 3).

**Table 3:** Variables associated with methicillin-resistant *Staphylococcus aureus *(MRSA) and vancomycin-resistant enterococcus (VRE) acquisition

Variable	Odds ratio ^a ^(95% CI)	*P *Value
**MRSA**		
MSSA carrier	0.52 (0.29, 0.95)	0.03
Intubation	4.65 (1.77, 12.26)	0.002
Fluoroquinolone	1.91 (1.20, 3.04)	0.01
ICU admit to negative swab		< .0001
1 day	1.0, reference	
2 days	1.97 (1.17, 3.30)	
≥ 3 days	15.59 (8.40, 28.94)	
**VRE**		
VSE carrier	1.37 (0.54, 3.48)	0.51
End-stage renal disease	2.60 (1.19, 5.70)	0.02
Albumin < 2	2.07 (1.12, 3.83)	0.02
Fluoroquinolone	1.90 (1.14, 3.17)	0.01
Third generation Cephalosporin	1.89 (1.15, 3.10)	0.01
ICU admit to negative swab		< .0001
1 day	1.0, reference	
2 days	1.42 (0.79, 2.56)	
≥ 3 days	15.13 (7.86, 29.14)	

^a^Based on generalized linear mixed model testing

CI, confidence interval.

Variables associated with VRE acquisition in bivariate testing are shown in Table 2. Similar to MRSA, increased time from ICU admission to initial VRE-negative swab, intubation, presence of a central line, and fluoroquinolone utilization were significantly associated with VRE acquisition. Several other factors were also associated with VRE acquisition in bivariate testing, including end-stage renal disease, wounds, rashes, low albumin, elevated creatinine, colonization pressure and macrolide, aminoglycoside, third-generation cephalosporin and carbapenem utilization. Many of these variables remained associated with VRE acquisition in multivariate testing using generalized linear mixed models (Table 3). However, in contrast to MRSA, VSE carriage was not associated with VRE acquisition.

## Discussion

Among ICU patients from a tertiary care medical center, we show that MSSA carriage results in a 50% reduced odds of MRSA acquisition when extensively accounting for other risk factors. These results support the concept that various *S. aureus *strains compete for occupancy of the anterior nares [40]. In this case, the protective nature of MSSA likely arises from being the initial occupant of the niche. It is likely that the presence of MRSA would similarly prevent the establishment of MSSA in the anterior nares. Thus, the presence or absence of the *mec A *gene alone is used as a surrogate means to distinguish *S. aureus *strains, rather than to suggest a competitive advantage in the absence of beta-lactam antibiotics.

However, regardless of the mechanism for competition between strains, evidence for competition supports the need to be judicious in applying decolonization regimens to eradicate the *S. aureus *reservoir. In particular, MSSA carriage may be preferable to the chance for re-colonization with an MRSA strain in certain high risk patient populations since it has been suggested in several studies that MRSA infections produce greater morbidity, mortality, and cost compared to MSSA infections in case mix-adjusted patient populations [2,3,16-18,41,42].

These results are relevant to decolonization strategies that are increasingly used and have been shown to successfully reduce MSSA and MRSA infections among carriers in high-endemic settings, including intensive care units and patients undergoing surgical procedures [43-51]. Currently, cardiac surgeons have a national guideline for pre-operative screening and decolonization of *S. aureus *to reduce *S. aureus *surgical site infections [43-45]. Increasingly, chlorhexidine and mupirocin are being routinely applied to MRSA carriers in hospitals to reduce healthcare-associated MRSA infection [46,49,52,53].

The large body of evidence demonstrating substantial benefits of decolonization should be weighed against the potential of increased MRSA acquisition risk due to a vacated anterior nares niche. Studies are needed to evaluate whether decolonization of MSSA carriers increases the risk of acquiring MRSA when discharging patients to high-endemic MRSA healthcare settings. On the other hand, we recognize this risk may be mitigated by decolonization which could reduce the prevalence and transmission of MRSA in post-discharge healthcare settings.

Consistent with prior reports, we found that patients who acquired MRSA and VRE had longer ICU lengths of stay [35,54,55]. We controlled for comorbidities and procedures that may have accounted for this and identified mechanical ventilation, fluoroquinolone exposure, and increased ICU duration prior to the initial negative swab as independent predictors of MRSA acquisition [21,35,56-58]. We also assessed other previously defined risk factors, but we highlight the protective nature of MSSA when performing a comprehensive evaluation of potential factors associated with MRSA acquisition. Our work indicates that interactions between colonizing *S. aureus *strains should be considered when evaluating patient-level predictors of MRSA acquisition, particularly in the setting of decolonization therapy.

In contrast to the association between MSSA and MRSA colonization, VSE was not protective against VRE acquisition. This latter finding is consistent with the abundance of microbial flora in the gut reservoir, where antibiotic-susceptible and resistant strains are not mutually exclusive for intestinal colonization. Similar to prior papers, we identified several risk factors associated with VRE acquisition including end-stage renal disease, active wounds, and low serum albumin levels [59,60].

Our study has important limitations. First, this study was restricted to ICU patients from a tertiary care hospital, and nearly 90% of our study population underwent surgery. Due to differences in patient populations, our findings may not be generalizable to other hospitals or non-ICU settings. Second, our work was often reliant on either nares or rectal screening alone to determine MRSA or VRE acquisition, respectively. If the sensitivity of these single-site screening tests was low, some of our controls may have actually harbored MRSA or VRE, and some of our cases may have actually been long term carriers. Nevertheless, this would have reduced the differences found between the groups. Finally, our results may not be generalizable to MRSA clones that do not predominantly colonize the anterior nares. While this has been suggested for community-associated clones, other research has found no difference in the strain-specific distribution of body site carriage among nursing home residents [61].

## Conclusions

We found that MSSA nasal carriage conferred a 50% reduction in the odds of MRSA acquisition among ICU patients. In contrast, no protective effect was observed for VSE. These findings are important for decolonization regimens that may eliminate MSSA and predispose to MRSA acquisition in high-endemic settings, such as ICUs, nursing homes, and rehabilitation centers. Additional studies are needed to better understand the degree to which MSSA is protective and the long-term impact of decolonization relative to subsequent healthcare exposures.

## Key messages

• MSSA carriage significantly protects against MRSA acquisition in ICUs.

• In contrast, VSE carriage does not protect against VRE acquisition in ICUs.

• Studies are needed to evaluate whether decolonization of MSSA ICU carriers increases the risk of acquiring MRSA when discharging patients to high-endemic MRSA healthcare settings.

## Abbreviations

ICU: intensive care unit; MRSA: methicillin-resistant *Staphylococcus aureus; *MSSA: methicillin-sensitive *Staphylococcus aureus; *VRE: vancomycin-resistant enterococci; VSE: vancomycin-sensitive enterococcus.

## Competing interests

The authors declare that they have no competing interests.

## Authors' contributions

JL participated in the study design and coordination. HP performed data collection and contributed to the study design. SRS and KK participated in the study conception and design and performed the statistical analysis. RD contributed to the data interpretation and manuscript preparation. SH and RP conceived of the study, contributed to its design and analysis, and critically revised the manuscript for intellectual content. All authors read and approved the final manuscript.
